# miRNA-Dependent Regulation of AKT1 Phosphorylation

**DOI:** 10.3390/cells11050821

**Published:** 2022-02-26

**Authors:** Mallory I. Frederick, Tarana Siddika, Pengcheng Zhang, Nileeka Balasuriya, Matthew A. Turk, Patrick O’Donoghue, Ilka U. Heinemann

**Affiliations:** 1 Department of Biochemistry, The University of Western Ontario, 1151 Richmond Street, London, ON N6A 5C1, Canada; mfreder8@uwo.ca (M.I.F.); tsiddika@uwo.ca (T.S.); pzhan33@uwo.ca (P.Z.); bbalasur@uwo.ca (N.B.); mturk5@uwo.ca (M.A.T.); patrick.odonoghue@uwo.ca (P.O.); 2 Department of Chemistry, The University of Western Ontario, 1151 Richmond Street, London, ON N6A 5C1, Canada

**Keywords:** miRNA, posttranslational modification, protein phosphorylation, oncogenic kinase, signaling

## Abstract

The phosphoinositide-3-kinase (PI3K)/AKT pathway regulates cell survival and is over-activated in most human cancers, including ovarian cancer. Following growth factor stimulation, AKT1 is activated by phosphorylation at T308 and S473. Disruption of the AKT1 signaling pathway is sufficient to inhibit the epithelial-mesenchymal transition in epithelial ovarian cancer (EOC) cells. In metastatic disease, adherent EOC cells transition to a dormant spheroid state, characterized previously by low S473 phosphorylation in AKT1. We confirmed this finding and observed that T308 phosphorylation was yet further reduced in EOC spheroids and that the transition from adherent to spheroid growth is accompanied by significantly increased levels of let-7 miRNAs. We then used mechanistic studies to investigate the impact of let-7 miRNAs on AKT1 phosphorylation status and activity in cells. In growth factor-stimulated HEK 293T cells supplemented with let-7a, we found increased phosphorylation of AKT1 at T308, decreased phosphorylation at S473, and enhanced downstream AKT1 substrate GSK-3β phosphorylation. Let-7b and let-7g also deregulated AKT signaling by rendering AKT1 insensitive to growth factor simulation. We uncovered let-7a-dependent deregulation of PI3K pathway components, including PI3KC2A, PDK1, and RICTOR, that govern AKT1 phosphorylation and activity. Together, our data show a new role for miRNAs in regulating AKT signaling.

## 1. Introduction

Epithelial ovarian cancer (EOC) has a 5-year relative survival rate of only 44%, making it the fifth most lethal cancer among women, largely attributed to high rates of metastasis. EOC metastasis begins when tumor cells are shed into the peritoneal cavity where they exist in suspension as single cells or multicellular aggregates known as spheroids. As spheroids, EOC cells survive in a dormant state, evading apoptosis and enabling metastasis. EOC cells utilize the phosphoinositide 3-kinase (PI3K)/AKT/mTOR pathway to dynamically regulate the dormant-to-proliferative metastatic switch. 

The PI3K/AKT/mTOR pathway controls the phosphorylation and thus activation of the three AKT isoforms (protein kinase B, PKB), AKT1, AKT2, and AKT3 [1], and their downstream targets, where increased AKT phosphorylation and activity are major contributors to EOC pathogenicity [2,3]. Indeed, AKT signaling is one of the most over-activated pathways in human cancers. Overexpression of the AKT isoforms is associated with multiple human cancers and hyper-activated AKT is a hallmark of > 50% of human tumors [4]. Surprisingly, despite their close sequence similarity, AKT1/2/3 have been shown to assume different roles, especially in cancer development. For example, in mice, knockdown of AKT2 or AKT3 isoforms increases ovarian cancer metastasis and tumor size, while AKT1 knockdown decreased both [5]. Here, we focus on the AKT1 kinase in the context of EOC. The AKT1 kinase stimulates numerous cellular signaling pathways, including networks promoting cell survival and inhibiting apoptosis. AKT1 is a leading drug target for EOC and other cancers where AKT1 phosphorylation status is linked to poor survival outcomes in patients [3].

**Figure 1:**
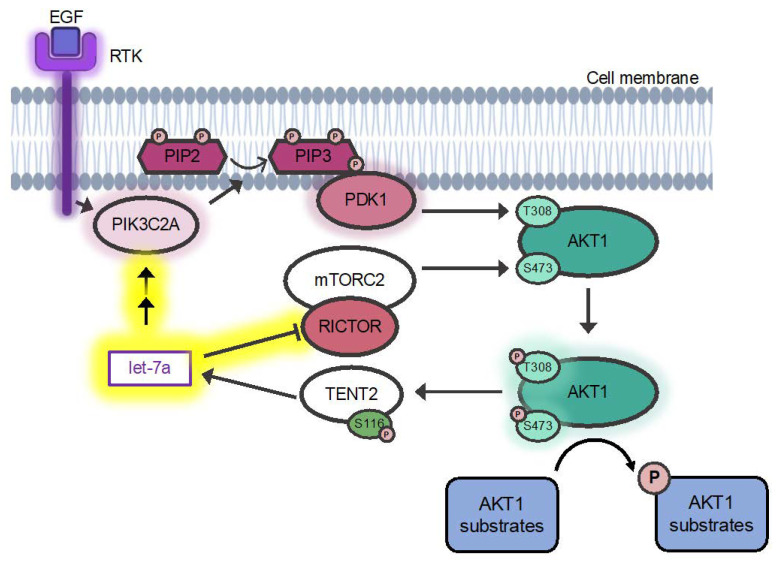
miRNA let-7a and epidermal growth factor stimulation control AKT1 phosphorylation by regulating PIK3C2A and RICTOR. Overview of the PI3K/AKT pathway and contributions of let-7a. Receptor tyrosine kinases (RTKs) are activated by epidermal growth factor (EGF) stimulation, leading to activation of PIK3C2A. Active PIK3C2A phosphorylates PtdIns (4,5) P2 (PIP2), converting it to PtdIns (3–5) P3 (PIP3). PIP3 recruits PDK1 and AKT1 to the cell membrane, leading to activation of PDK1, which subsequently phosphorylates AKT1 at Thr-308, activating the kinase. Fully activated AKT1 is formed by the additional phosphorylation of Ser-473 by mTORC2 and phosphorylates various cellular substrates. The AKT1 substrate TENT2 is inactivated by Ser-116 phosphorylation. Arrows indicate positive regulation, and flatheads indicate inhibition. Glows indicate proteins activated upon EGF stimulation. The established AKT pathway (black arrows) are shown along with the novel signaling pathway we identified (yellow highlights), where miRNA let-7a, a substrate of TENT2, forms a feedback mechanism wherein it inhibits the mTORC2 member RICTOR and increases PIK3C2A, leading to changes in downstream signaling.

AKT1 activity is regulated by phosphorylation at two key sites, T308 and S473 (Figure 1). The phosphorylation status at each site is a widely used diagnostic marker in cancers. Phosphorylation of T308 alone leads to ~400-fold increase in kinase activity [6]. Phosphorylation at S473 can provide additional activation of the kinase and regulates substrate selectivity of the activated kinase [7]. Cellular AKT1 activity is stimulated by insulin or growth-factor dependent phosphorylation. T308 is phosphorylated by the phosphoinositide-dependent kinase (PDK1) [6,8] and S473 by the mammalian target of rapamycin complex 2 (mTORC2) (Figure 1) [4,9]. Dephosphorylation and consequent inactivation of AKT1 are catalyzed by the protein phosphatase 2 (PP2A) at T308 and by the PH domain leucine-rich repeat protein phosphatase (PHLPP) at S473 [10,11]. As a result of extensive biochemical and cell-based studies, the mechanistic basis of AKT1 activation is coming into focus; however, the full range of processes that regulate or are regulated by AKT1 activity remain unknown. 

Multiple recent studies are establishing new connections between miRNAs and AKT1 signaling. AKT1 is a target of different miRNAs that regulate AKT1 protein levels in cancer cells. Binding of miRNA-422a to the 3′-untranslated region (UTR) of AKT1 leads to decreased production of AKT1, resulting in reduced tumor growth and proliferation in colorectal cancer cells. Conversely, down-regulation of miR-422a accelerates tumor growth by releasing its regulation of the PI3K/AKT pathway [12]. Studies in adult malignant gliomas showed that suppression of miRNA-637 induces cell invasion by releasing its interaction with the 3′-UTR of AKT1. The opposite effect was observed upon up-regulation of miR-637 expression, which inhibits proliferation, migration, and invasion of brain tumor cells [13]. In mesenchymal stem cells, AKT1 is downregulated by miRNA-149, where decreased miR-149 causes differentiation by increasing AKT1 expression [14]. 

MiRNAs and their regulatory pathways have emerged as new therapeutic targets for drug-resistant cancers. The conserved miRNA let-7a is a known regulator of oncogenesis that suppresses tumor proliferative activities by repressing oncogenic signaling pathways. Verified targets of let-7a include the oncogenes c-Myc, Rat sarcoma virus (Ras), high-mobility group A (HMGA), Janus protein tyrosine kinase (JAK), signal transducer and activator of transcription 3 (STAT3), and novel Np95/ICBP90-like RING finger protein (NIRF) [15]. Let-7a homeostasis is also stringently controlled, and maintenance of high let-7a abundance prevents unregulated cell proliferation [15]. The let-7a miRNA is a known tumor suppressor [15] that is downregulated in both established cell-based models of cancer and patient tumor samples, including in neuroblastomas, colon, and gastric cancers [16,17,18,19,20,21].

Motivated by the above studies that established the ability of miRNAs to regulate the AKT1 pathway as well as those documenting the association of let-7a with tumor cells, we hypothesized that let-7 family miRNAs may act to deregulate PI3K/AKT signaling. Thus, we investigated the impact of miRNAs from the let-7 family on AKT1 phosphorylation status and activity. We identified an inverse relationship between let-7a levels and AKT activity in adherent and spheroid EOC cells that we used as a well-established model of EOC metastasis. We also found AKT1 signaling was deregulated in EGF-stimulated cells supplemented with let-7 miRNAs, which were characterized by enhanced AKT1 signaling, increased phosphorylation at T308, and decreased phosphorylation at S473 of AKT1. Finally, we showed that let-7a deregulates the PI3K pathways components PIK3C2A and RICTOR to control AKT1 phosphorylation status. These data are the first report of let-7a-dependent regulation of AKT1 phosphorylation at both key regulatory sites.

## 2. Materials and Methods

### 2.1. Mammalian Cell Culture

HEK 293T cells and COS-7 cells were grown in Dulbecco’s Modified Eagle Medium (DMEM) supplemented with 10% fetal bovine serum (FBS) and 1% penicillin/streptomycin. TOV-21G cells were grown in DMEM:F12 supplemented with 10% FBS and 1% penicillin/streptomycin. All cells were grown at 37 °C in a 5% CO_2_ chamber. TOV-21G plates at 70–90% confluency were split into either standard tissue culture-treated plastic (adherent) or ultra-low attachment (ULA, spheroid) plates at the same dilution. Cells were grown on standard or ULA plates for 3 days before harvesting. Spheroid cultures were centrifuged to pellet cells and washed once with HBSS before trypsinization. Adherent cultures were harvested by standard trypsinization procedures. All washes and incubations were maintained for the same period between adherent and spheroid cultures, including trypsin treatment. Cell pellets washed with PBS were stored in −80 °C until RNA or protein extraction.

### 2.2. Transfections

Sixty femtomoles of miRNA let-7a/b/g (Supplementary Table S1) was used for transient transfections of HEK 293T cells grown to approximately 70–90% confluency in 6-well plates with Lipofectamine 2000 or 3000 (Thermo Fisher Scientific) for 24 h. HEK 293T cells transfected with mCherry-AKT1 were co-transfected with let-7a, let-7b, or no miRNA control, and 5 ng plasmid DNA for 24 h before EGF stimulation. For EGF stimulation experiments, cells were treated with 50 ng/µL human EGF (Sigma-Aldrich) for 15 min at room temperature before harvesting. COS-7 cells were co-transfected with mCherry-AKT1 and let-7a as above and serum starved for 4 h before EGF stimulation. Cells pellets were frozen at −80 °C until RNA or protein extraction.

### 2.3. RT-qPCR

Total RNA was extracted from mammalian samples using miRNAeasy Mini Kit (Qiagen). Primers for reverse transcription and quantitative PCR are listed in Supplementary Table S1. Reverse transcription of small RNAs was performed as described [22]. Quantitation of small RNAs and RT-qPCR of mRNAs was performed as described [23]. Briefly, 0.5–2 µg of total RNA was reverse transcribed using the High-Capacity cDNA Reverse Transcription Kit (Thermo Fisher). Amplification of cDNA was measured on Viia7 or QuantStudio 3 Real-Time PCR machines (Thermo Fisher) by Power Up or Power Track SYBR Green Master Mix (Thermo Fisher) fluorescence.

### 2.4. Protein Extraction

Cell pellets were resuspended in ice-cold lysis buffer 1 (50 mM Tris-HCl (pH 7.4), 1% Triton X-100, 150 mM NaCl, and 0.1% sodium dodecyl sulfate (SDS) supplemented with 1 mM phenylmethylsulfonyl fluoride (PMSF)) or ice-cold lysis buffer 2 (50 mM Na_2_HPO_4_, 1 mM Na_4_P_2_O_7_, 20 mM NaF, 2 mM EDTA, 2 mM EGTA, and 1% Triton X-100 supplemented with 300 µM PMSF, 1 mM dithiothreitol, and protease inhibitor cocktail (Roche)) and incubated on ice for ~10 min with vortexing. Lysates were clarified by centrifugation at 14,800× *g* for 10 min at 4 °C. Protein concentration was measured by Bradford, and equal amounts were loaded for each sample. 

### 2.5. Western Blotting

Cell lysates were diluted to equal concentrations and 10–100 µg of total protein was loaded on 8% or 12% polyacrylamide gels, depending on the molecular weight of the protein of interest. Gels were run for approximately 30 min at 90 V and 1–3 h at 120–140 V, until adequate separation was achieved based on ladder migration. Proteins were transferred to PVDF membrane activated in methanol using BioRad Trans-Blot Turbo Transfer System. Membranes were subsequently blocked in 5% BSA TBST for 1 h at room temperature, then in primary antibodies diluted in 5% BSA TBST overnight at 4 °C or for 1–4 h at room temperature. Washes consisted of 3 × 10 min in 1% BSA TBST followed by incubation in secondary antibodies diluted in 1% BSA TBST for 1–2 h at room temperature and final washes of 3 × 5 min in TBST. Membranes were stored in TBS until imaged by fluorescence or chemiluminescence. For blots performed using fluorescent secondary antibodies, all steps beginning with the addition of the secondary antibody were performed in the dark. Antibodies are listed in Supplementary Table S2.

### 2.6. Quantification and Statistical Analysis

At least 3 replicates were used for all experiments, with individual data points indicated on all graphs. Graphs were produced in GraphPad Prism. Western blots were quantified using Image Lab (Bio-Rad), and data were compiled and normalized in Microsoft Excel. RT-qPCR data were analyzed by the ΔΔCt method in Microsoft Excel. *p*-values were calculated by *t*-test, one-way ANOVA, or two-way ANOVA as appropriate in GraphPad Prism. *p*-values are indicated by asterisks where ns = not significant, * *p* < 0.05, ** *p* < 0.01, *** *p* < 0.001, **** *p* < 0.0001.

## 3. Results

### 3.1. AKT1 Phosphorylation and let-7 Abundance in Epithelial Ovarian Cancer Cells

EOC cells are a well-studied model system for AKT1-driven cancers. The dormant-to-proliferative metastatic switch in EOC cells is directed by changes in AKT1 phosphorylation status [2,3]. Cells aggregate as dormant spheroids and are signified by low AKT1 phosphorylation at S473. Inhibition of AKT1 activation prevents the switch from dormant status to proliferative cells [2,3], where high phosphorylation at S473 is required. 

**Figure 2:**
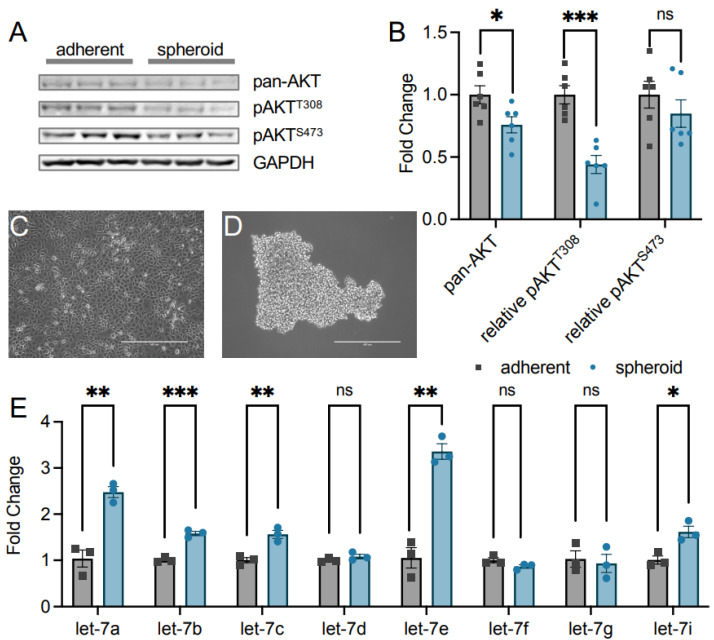
AKT phosphorylation and let-7 miRNA levels are inversely correlated in epithelial ovarian cancer metastasis. (**A**) Western blots of pan-AKT and pAKT protein levels were (**B**) quantified from adherent (grey squares) and spheroid (blue circles) TOV-21G ovarian cancer cells. Representative images of TOV-21G epithelial ovarian cancer (EOC) cells grown as (**C**) adherent and (**D**) spheroid cultures for 72 h. Scale bars indicate 400 nm. (**E**) RT-qPCR of let-7 family miRNAs in adherent (grey squares) and spheroid (blue circles) TOV-21G EOC cells. The data include *n* = 3 or *n* = 6 biological replicates. Error bars show ± 1 SEM. Asterisks indicate significance (ns, not significant; * *p* < 0.05; ** *p* < 0.01, *** *p* < 0.001).

We tested the AKT abundance and phosphorylation status (Figure 2A,B) in both adherent (Figure 2C) and spheroid cells (Figure 2D) from the TOV-21G EOC cell line. We found that the overall pan-AKT abundance of all AKT1/2/3 isoforms was significantly reduced to 0.76-fold in spheroid cells (Figure 2A,B). Due to this change, we calculated changes in relative pan-AKT T308 and S473 phosphorylation and found T308 was significantly reduced to 0.44-fold in spheroids while S473 was unchanged (Figure 2A,B). Decreased phosphorylation of S473 was reported previously [2], and while we observed this effect, we now show that this is a function of reduced AKT levels in TOV-21G cells, with T308 phosphorylation status even more greatly reduced in EOC spheroid cells compared to adherent cells. 

The let-7 miRNA family is a well-known suppressor of cancer and metastasis [15], prompting us to investigate let-7 miRNA abundance in both spheroid and adherent cells using quantitative PCR. Interestingly, TOV-21G spheroid cells have a significantly increased abundance of multiple let-7 family miRNAs (Figure 2E). miRNAs let-7a and let-7e were the most dramatically increased by 2.5- and 3.4-fold in spheroid cells, respectively. Let-7b, let-7c, and let-7i were each significantly increased by 1.6-fold in the dormant spheroids. The miRNAs let-7d, let-7f, and let-7g did not change in abundance in spheroids relative to adherent cells. This correlation between increased let-7 miRNA levels and decreased AKT phosphorylation status indicates a novel signaling axis linking let-7 miRNAs to AKT phosphorylation status in these cells. 

### 3.2. Let-7a miRNA Does Not Control AKT1 Abundance 

**Figure 3:**
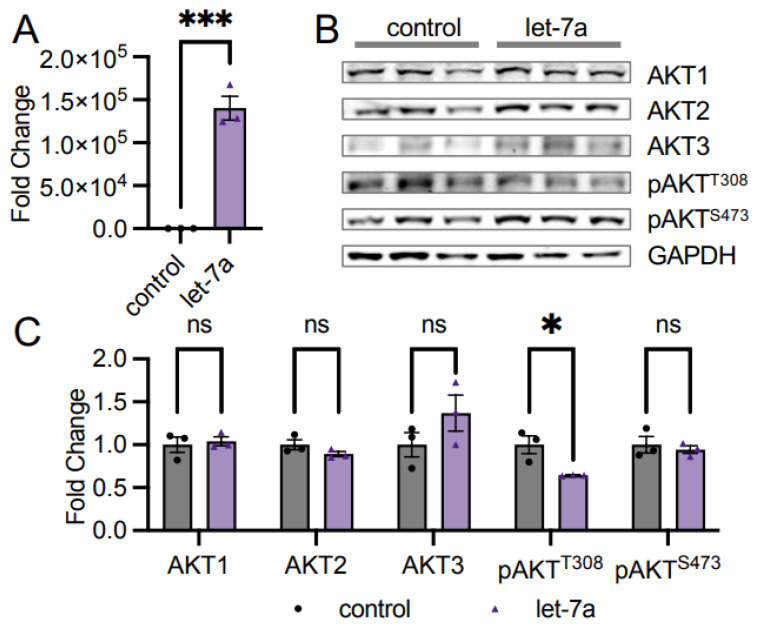
AKT phosphorylation is downregulated in let-7a transfected cells. (**A**) RT-qPCR of let-7a miRNA levels in unstimulated HEK 293T cells transfected with 60 fmol let-7a (purple triangles) or a control transfection lacking RNA (black circles). (**B**) Western blots of AKT isoforms and pAKT protein levels were (**C**) quantified in cells treated as in (**A**). The data include *n* = 3 biological replicates. Error bars show ± 1 SEM. Asterisks indicate significance (ns, not significant; * *p* < 0.05; *** *p* < 0.001).

As previous literature shows multiple miRNAs impacting AKT protein abundance [12,13,14], we speculated that the observed decrease in pan-AKT levels in high let-7 conditions in EOC spheroids (Figure 2) may be related to those miRNAs. We measured AKT levels in cells supplemented with let-7a to investigate this relationship, as there was a large change in expression (Figure 2) and let-7a has been previously associated with AKT signaling in cancers [24,25]. We transfected HEK 293T cells with the miRNA let-7a, confirmed transfection by RT-qPCR (Figure 3A), and measured AKT1, AKT2, and AKT3 protein abundance using western blotting. Transfection with let-7a did not alter AKT1 abundance (Figure 3B,C). The result was anticipated, as the AKT1 3′UTR does not encode known let-7a binding sites according to miRDB [26,27]. We further observed no change in the abundance of the AKT2 or AKT3 isoforms (Figure 3B,C), corroborating that let-7a does not control AKT abundance. Interestingly, phosphorylation at T308 significantly decreased to 0.64-fold in let-7a transfected cells relative to control cells, while S473 phosphorylation was unchanged (Figure 3B,C), indicating that let-7a disrupts the pathways leading to the phosphorylation of T308 in unstimulated cells.

### 3.3. Let-7 miRNAs Regulate AKT Phosphorylation

#### 3.3.1. Let-7a Increases AKT1 Phosphorylation at T308 

As the phospho-specific antibodies for AKT sites T308 and S473 do not sufficiently distinguish between the three AKT isoforms, we decided to use an established mCherry-AKT1 overexpression construct [6], allowing us to blot for both phosphorylation sites and measure changes in AKT1 phosphorylation specifically due to the increased relative molecular weight of the fusion protein. This construct is visible as two high molecular weight bands separate from endogenous AKT1/2/3, where the larger corresponds to the full-length protein and the smaller a partial degradation of the mCherry tag, as reported previously [6]. To elucidate the potential of let-7a to regulate factors that are upstream of AKT1 phosphorylation, we conducted AKT1 overexpression experiments in unstimulated and EGF-stimulated cells. Cells overexpressing the mCherry-AKT1 fusion protein were co-transfected with let-7a and subsequently stimulated with EGF. 

**Figure 4:**
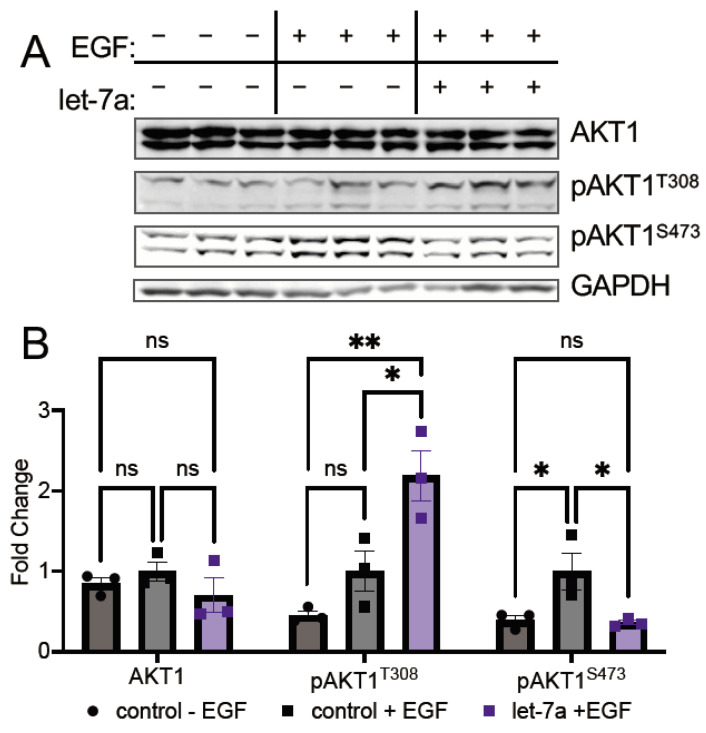
let-7a deregulates AKT1 phosphorylation in epidermal growth factor stimulated cells. (**A**) Western blots and (**B**) quantification of mCherry-AKT1 and mCherry-pAKT1 levels in HEK 293T cells co-transfected with 5 µg mCherry-AKT1 plasmid and 60 fmol let-7a (purple) or a control lacking RNA (black) for 24 h and stimulated with epidermal growth factor (EGF, square symbols) or without (circle symbols) EGF for 15 min. The data include *n* = 3 biological replicates. Error bars show ± 1 SEM. Asterisks indicate significance (ns, not significant; * *p* < 0.05; ** *p* < 0.01).

In HEK 293T cells, as expected [28], EGF stimulation increased S473 phosphorylation on mCherry-AKT1 in comparison to unstimulated cells (Figure 4A,B). Interestingly, transfection of let-7a in combination with EGF stimulation leads to a significant 2.5-fold reduction in S473 phosphorylation relative to EGF-stimulated control (Figure 4A,B), revealing an unexpected link between let-7a activity and AKT1 S473 phosphorylation in highly proliferating cells, and corroborating the inverse relationship let-7 and pS473 levels observed in EOC cells. Interestingly, at T308, the opposite effect was observed: let-7a transfection in combination with EGF stimulation led to significant hyper-phosphorylation at T308 by 2.2-fold over EGF stimulation alone (Figure 4A,B). In contrast, let-7a transfection showed no effect on mCherry-AKT1 phosphorylation in the absence of EGF stimulation (Supplementary Figure S1). The combinatorial effect of let-7a and EGF leads to hyper-phosphorylation on the primary activating site in AKT1. Thus, let-7a in proliferating cells may increase AKT1 activity by increasing phosphorylation at T308 while simultaneously modulating substrate selectivity linked to S473 phosphorylation. 

#### 3.3.2. Let-7a Regulation of T308 Causes Downstream Changes in AKT1 Signaling

**Figure 5:**
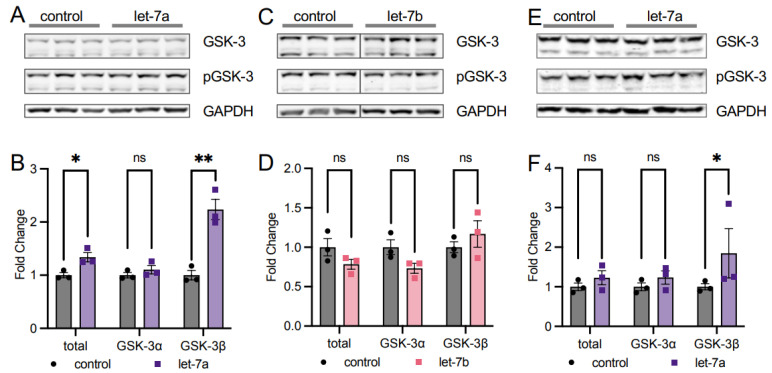
let-7a activation of AKT1 increases downstream phosphorylation of the substrate GSK-3. (**A**) Western blots and (**B**) quantification of GSK-3 and pGSK-3 in HEK 293T cells co-transfected with 60 fmol let-7a (purple squares) or a control lacking RNA (black circles) and mCherry-AKT1 for 24 h and stimulated with epidermal growth factor (EGF) for 15 min. (**C**) Western blots and (**D**) quantification of GSK-3 and pGSK-3 in HEK 293T cells co-transfected 60 fmol let-7b (pink squares) or a control lacking RNA (black circles) and mCherry-AKT1 for 24 h and stimulated with epidermal growth factor (EGF) for 15 min. (**E**) Western blots and (**F**) quantification of GSK-3 and pGSK-3 in COS-7 cells co-transfected with 60 fmol let-7a (purple squares) or a control lacking RNA (black circles) and mCherry-AKT1 for 24 h and stimulated with epidermal growth factor (EGF) for 15 min. Note that all graphs show relative phosphorylation of the AKT substrate GSK-3 in respective experimental conditions. The data include *n* = 3 biological replicates. Error bars show ± 1 SEM. Asterisks indicate significance (ns, not significant; * *p* < 0.05, ** *p* < 0.01).

Since our data show that let-7a increases T308 phosphorylation but decreases S473 phosphorylation of AKT1 in stimulated cells, we investigated the overall impact on AKT1 activity by examining the downstream phosphorylation of a known AKT1 target. We measured the phosphorylation status of the AKT1 substrate glycogen synthase kinase-3 (GSK) [29] in HEK 293T cells. In EGF-stimulated and let-7a transfected cells overexpressing mCherry-AKT1, phosphorylation of GSK-3β was increased significantly by 2.2-fold compared to EGF-stimulated cells alone (Figure 5A,B). Although both the alpha and beta isoforms are AKT1 targets, we observed this effect specific to the GSK-3β isoform, with no effect of let-7a on GSK-3α phosphorylation in stimulated cells. We also measured GSK-3 phosphorylation in AKT1-overexpressing HEK 293T cells transfected with let-7b and found no change in GSK-3 phosphorylation status (Figure 5C,D). The data are further evidence that T308 phosphorylation is critical for AKT1 activity and downstream signaling in human cells and they reveal a novel and specific role for let-7a in regulating AKT1 signaling. 

Similar to HEK 293T cells, COS-7 cells stimulated with EGF and transfected with let-7a showed the same significant and specific increase in relative GSK-3β phosphorylation (Figure 5E,F), indicating that the let-7a regulatory axis linked to AKT signaling functions in multiple mammalian cell lines. Previous studies showed that phosphorylation at T308 is necessary and sufficient for maximal AKT1 signaling in cells [7,30] regardless of the S473 phosphorylation status. Our data confirm the increase in T308 phosphorylation alone is sufficient to increase downstream AKT1 signaling. 

### 3.4. Let-7b and Let-7g Also Counteract EGF Stimulation at S473

**Figure 6:**
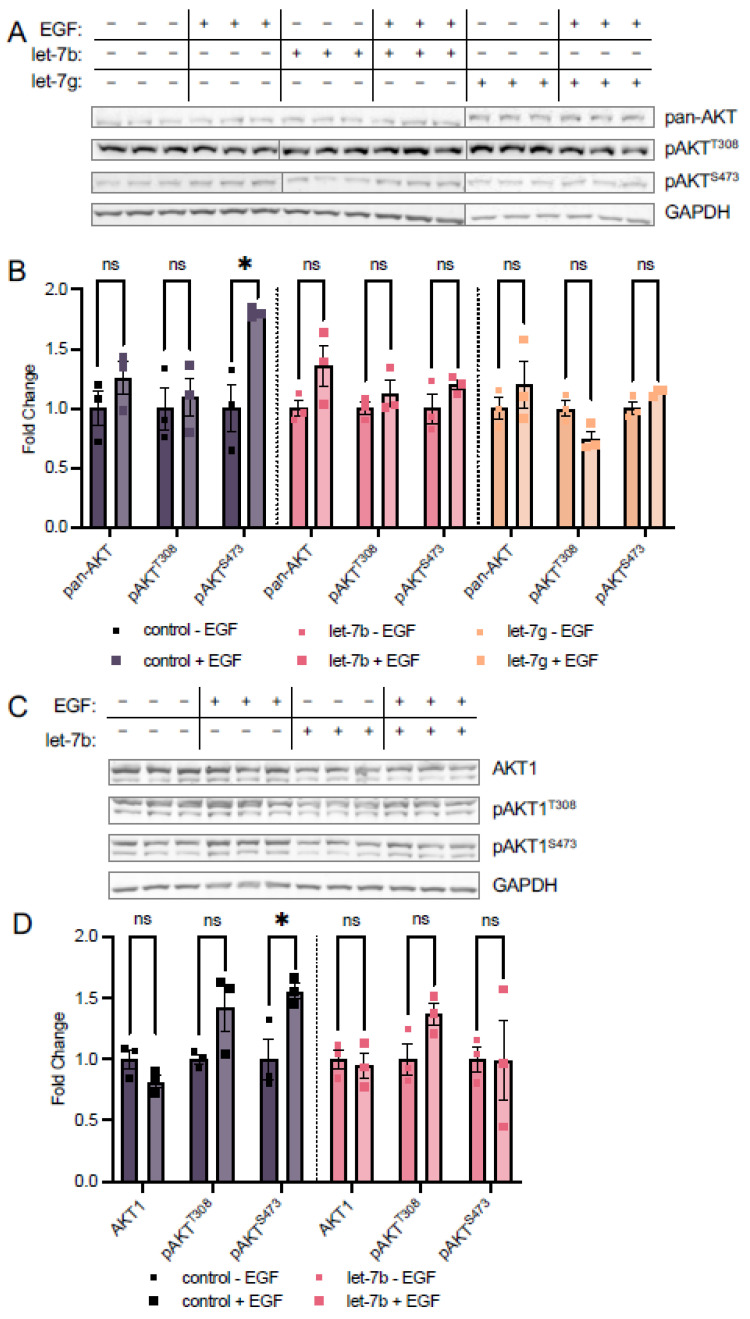
let-7b and let-7g regulate phosphorylation of AKT at Ser-473. (**A**) Western blots and (**B**) quantification of pan-AKT and pAKT protein levels in HEK 293T cells transfected with 60 fmol let-7b (pink), let-7g (orange), or no RNA control (grey) for 24 h and stimulated with epidermal growth factor (EGF, square symbols) or without (circle symbols) EGF for 15 min. (**C**) Western blots and (**D**) quantification of mCherry-AKT1 and mCherry-pAKT1 levels in HEK 293T cells co-transfected with 5 µg mCherry-mAKT1 plasmid and 60 fmol let-7b (pink) or a control lacking RNA (grey) for 24 h and stimulated with (square symbols) or without (circle symbols) EGF for 15 min. The data include *n* = 3 biological replicates. Error bars show ± 1 SEM. Asterisks indicate significance (ns, not significant; * *p* < 0.05).

In addition to let-7a, we measured the expression of the let-7 miRNA family in TOV21G EOC cells and found upregulation of let-7b in spheroids relative to adherent cells, though to a lesser extent than let-7a, while let-7g was unchanged (Figure 2E). To assess the potential roles of these miRNAs in regulating AKT1 phosphorylation and address the spectrum of let-7 miRNA expression in EOC metastasis, we transfected let-7b and let-7g into HEK 293T cells. We first measured the effects of these two miRNAs on phosphorylation of endogenous AKT using antibodies targeting pan-AKT phosphorylation and found that while EGF stimulation caused a significant 1.8-fold increase in pan-AKT phosphorylation at S473 in control cells, S473 phosphorylation was unresponsive to EGF treatment in both let-7b and let-7g transfected cells (Figure 6A,B). However, unlike let-7a, neither let-7b nor let-7g affected T308 phosphorylation in either stimulated or unstimulated cells. These data indicate that the different let-7 family members play both overlapping and distinct roles in regulating AKT phosphorylation. 

To assess the impact of let-7b on AKT1 phosphorylation specifically, we combined let-7b miRNA transfection with mCherry-AKT1 plasmid transfection and EGF stimulation as above. Co-transfection of let-7b and mCherry-AKT1 led to decreased AKT1 expression, and we found let-7b also prevents the EGF-dependent increase in mCherry-AKT1 S473 phosphorylation. We observed no change in relative AKT1 S473 phosphorylation when let-7b transfected cells are stimulated, compared to a significant 1.6-fold increase in pAKT1^S473^ when control cells are EGF stimulated (Figure 6C,D). Surprisingly, let-7b did not affect T308 phosphorylation status in either stimulated or unstimulated conditions (Figure 6C,D), suggesting that deregulation of T308 phosphorylation is specific to let-7a. 

### 3.5. Regulation of AKT1 Phosphorylation

#### 3.5.1. A Mechanism for Let-7a Regulation of AKT1 Phosphorylation at T308 

**Figure 7:**
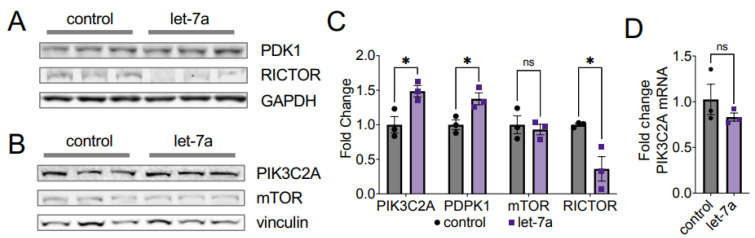
Let-7a deregulates AKT1 phosphorylation via PIK3C2A and RICTOR. (**A**,**B**) Western blots and (**C**) quantification of PDK1, RICTOR, PIK3C2A, and mTOR in HEK 293T cells transfected 60 fmol let-7a (purple squares) or a control lacking RNA (black circles) for 24 h. Cells in (**A**) were stimulated with EGF for 15 min. (**D**) RT-qPCR of PIK3C2A mRNA from cells transfected with 60 fmol let-7a (purple squares) or a control lacking RNA (black circles) for 24 h and stimulated with EGF for 15 min. The data include *n* = 3 biological replicates. Error bars show ± 1 SEM. Asterisks indicate significance (ns, not significant; * *p* < 0.05).

To show how let-7a impacts AKT1 phosphorylation status, we investigated the pathways regulating phosphorylation of the key sites T308 and S473. AKT1 T308 is phosphorylated by the upstream kinase PDK1. PDK1 itself is activated in a cascade following EGF stimulation and phosphorylation of a receptor tyrosine kinase (RTK), which recruits phosphoinositide 3-kinases (PI3Ks) to the membrane. PI3Ks in turn phosphorylates the phosphatidylinositol 4,5-bisphosphate (PtdIns (4,5) P2; PIP2) to phosphatidylinositol (3,4,5)-trisphosphate (PtdIns(3,4,5)P3; PIP3), which activates AKT1 by recruiting both PDK1 and AKT1 to the membrane, leading to phosphorylation at T308 (Figure 1). In concordance with our finding of increased AKT1 T308 phosphorylation in let-7a supplemented and EGF-stimulated cells, we observed a coincident and significant 1.4-fold increase in PDK1 protein levels with let-7a plus EGF stimulation relative to EGF stimulation alone (Figure 7A,C). 

We next probed the abundance of the phosphatidylinositol-4-phosphate 3-kinase catalytic subunit type 2 alpha (PIK3C2A) in response to let-7a transfection. We observed a significant 1.5-fold increase in PIK3C2A protein levels in cells supplemented with let-7a (Figure 7B,C). No difference was observed on the mRNA level of *PIK3C2A* (Figure 7D), indicating that the regulation does not occur on the transcriptional level. The data indicate that let-7a supplemented cells increase the abundance of major kinases upstream of AKT1 T308, which results in stimulation of the PI3K/AKT1 pathway (Figure 1) and downstream signaling (Figure 6).

#### 3.5.2. Let-7 miRNAs Regulate AKT1 Phosphorylation at S473

AKT1 S473 is phosphorylated by the mammalian target of rapamycin (mTOR) complex 2, (mTORC2) [9], a seven-subunit protein complex, including the core components mTOR and RPTOR independent companion of mTOR complex 2 (RICTOR). S473 phosphorylation is catalyzed by mTORC2 upon growth factor stimulation (Figure 1) [4,6,9]. We probed the protein abundance of two of the mTORC2 subunits, RICTOR and mTOR. We found that the abundance of RICTOR, a confirmed let-7a target [24], significantly decreased by 2.5-fold following let-7a transfection in EGF-stimulated cells (Figure 7A,C). mTOR protein levels remained unchanged after let-7a transfection (Figure 7B,C). These data agree with the previous finding from gastric cancer cells that RICTOR is a direct target of let-7a [24], resulting in the decreased phosphorylation of AKT1 at S473 that we observed (Figure 4 and Figure 5). Given that the seed sequence of let-7a is shared between the let-7 family miRNAs, this reduction in RICTOR is likely responsible for the same defects we observed in S473 phosphorylation in cells treated with let-7b and let-7g. Our data support a new model wherein decreased mTORC2 formation due to let-7-dependent reduction in RICTOR (Figure 7) leads to an EGF-dependent decrease in AKT1 phosphorylation at S473 (Figure 1).

## 4. Discussion

The over-activation of the PI3K/AKT signal transduction pathway in response to extracellular signals is a crucial step leading to the progression of cancer and tumorigenesis [31,32]. While both regulatory phosphorylation sites in AKT1 (T308, S473) are associated with disease and used as clinical markers, in some cancers high levels of T308 phosphorylation are associated with poor prognosis [33], while in other cancers, poor survival correlates with high pS473 levels [34]. In addition, we [7] and others [35,36] previously showed that S473 phosphorylation modulates AKT1 substrate selectivity, presumably due to a disorder-to-order conformational shift in the AKT1 hydrophobic motif upon S473 phosphorylation [37]. Here, we discovered that AKT1 phosphorylation and activity are regulated by the let-7 family of miRNAs. In stimulated cells, let-7a promoted T308 phosphorylation while repressing S473 phosphorylation, which produced greater total AKT1 activity, as evidenced by increased phosphorylation of the AKT1 substrate GSK-3β. In let-7b and let-7g-transfected cells, a similar effect was seen in repression of S473 phosphorylation, while no changes in T308 phosphorylation or downstream signaling were observed. Therefore, let-7 family members have distinct roles in controlling AKT phosphorylation. 

### 4.1. Let-7 miRNAs in Ovarian Cancer Cells

The shift from adherent to spheroid cells during EOC metastasis has previously been reported to be AKT-dependent [2,38,39,40], and spheroid cells are characterized by low S473 phosphorylation levels on AKT [2,38]. We previously showed that T308 phosphorylation is the primary determinant for AKT1 activity [6,7,29]. Thus, we investigated total pAKT^T308^ levels of all isoforms in spheroids compared to adherent cells. We found TOV2-1G spheroids have reduced phosphorylation at T308 and reduced S473 is a function of reduced total AKT protein levels. This is the first report of reduced T308 phosphorylation in EOC spheroids.

Several let-7 family miRNAs change expression during cancer metastasis (reviewed in [41]), implicating a relationship between let-7 abundance and metastatic behavior. We here show increased abundance of several let-7a miRNAs in dormant TOV-21G spheroid cells compared to proliferating adherent cells. While EOCs have high rates of recurrence due to metastasis and resistance to platinum-based therapeutics, increases in let-7e [42] and let-7g [43] have both been linked to chemotherapy sensitization in EOC. Additionally, let-7b has been linked to chemotherapy sensitization in *KRAS* mutant cancer cell lines, where high let-7b alone had no effect but lead to decreased phospho-AKT levels when combined with chemotherapies [44]. The TOV-21G cell line contains an activating *KRAS* mutation. The combination of the findings we report here and previous studies investigating let-7 miRNAs in cancer environments indicate a broad role for this family in cancer progression and metastasis.

### 4.2. AKT Isoforms and Let-7a 

We here set out to investigate the negative correlation between let-7 abundance and AKT abundance, phosphorylation, and activity that we observed in EOC cells. In independent experiments, we transfected these three let-7 isoforms into HEK 293T cells. In HEK 293T cells, let-7a transfection alone does not alter the abundance of any of the three AKT isoforms. Although no let-7a binding sites are found in the AKT1 3′UTR, the 3′UTR of AKT2 mRNA encodes one potential let-7a binding site, and the 3′ UTR of AKT3 mRNA encodes three potential let-7a binding sites [26,27]. A previous study showed that let-7a indeed repressed AKT2 expression in papillary thyroid carcinoma cells [25], yet our data showed no significant change in protein abundance upon let-7a transfection in HEK 293T cells. Thus, let-7a repression of AKT2 translation may be cell-type specific. Indeed, the timing of let-7 expression is known to regulate developmental pathways leading to cell differentiation [45,46], and changes in let-7 levels were linked to the oncogenic transformation of neuroblasts to neuroblastomas [19]. As let-7a is a known regulator of many oncogenes, including Myc, Ras, HMGA, JAK, STAT3, and NIRF [15], our discovery that let-7 miRNAs regulate AKT1 phosphorylation makes them exciting new targets for the development of chemotherapeutics in AKT1-derived cancers. 

### 4.3. Let-7 miRNAs Regulate AKT1 Ser473 Phosphorylation via RICTOR

The S473 residue is located in the AKT1 hydrophobic motif, a regulatory loop distant from the active site [29,47]. In addition to phosphorylation at T308, phosphorylation at S473 leads to full activation of AKT1 on some substrates [6,7,48]. We recently showed that S473 phosphorylation modulates AKT1 activity in a substrate-dependent manner and increases AKT1 activity for only half of the known substrates [7]. 

AKT1 S473 is phosphorylated by the mTORC2 complex [9]. The mechanism of regulation of mTORC2 itself is not completely known. mTORC2 activation and association with the ribosome were shown to be growth factor dependent [49,50], and its activity relies on the scaffolding protein RICTOR [51]. Downregulation of RICTOR was previously shown to reduce pAKT^S473^ levels in gastric cancer cells, where let-7a negatively regulates translation of RICTOR mRNA [24]. Our data identify a similar mechanism in HEK 293T cells, where we show that increased let-7a levels downregulate RICTOR protein abundance, making the cells unable to respond to EGF stimulation and phosphorylate AKT1 at S473 (Figure 1). In summary, our data show that cells with increased let-7a, let-7b, or let-7g maintain pAKT^S473^ levels similar to control cells but are unable to respond to EGF stimulation due to reduced RICTOR levels.

Since S473 phosphorylation modulates AKT substrate selectivity [7,35,36], the ability of let-7 miRNAs to down-regulate pS473 may impact the phosphorylation level of AKT1 targets in a substrate-dependent manner. Although we investigated the phosphorylation of GSK-3β in response to let-7a and let-7b transfection, the complete set of cellular AKT1 targets and their individual responses to single or double phosphorylation status of AKT1 is yet to be fully elucidated [7], indicating that many other substrates may have changed phosphorylation status in addition to GSK-3β. Indeed, despite the decrease in S473 phosphorylation, GSK-3β phosphorylation increased with let-7a transfection, due to T308 hyper-phosphorylation. The stimulation of AKT signaling was let-7a specific as the same effect was not observed in let-7b transfected cells, where both T308 and GSK-3 phosphorylation of both isoforms remained unchanged. Increased GSK-3β phosphorylation is in accordance with previous biochemical data showing pAKT1^T308^ retains most of the activity of the doubly phosphorylated ppAKT1^T308,S473^ in phosphorylating GSK-3β [6]. The lack of change in GSK-3α phosphorylation may be reflective of the different cell type-specific roles of the two isoforms upon AKT activation [22]. The observation is also in agreement with previous research that indicated AKT2 is the main AKT isoform responsible for GSK-3α phosphorylation rather than AKT1 [23]. Future work will investigate the defective S473 phosphorylation we observed in let-7-supplemented cells and its downstream impacts on the phosphorylation status of the pool of AKT1 substrates.

### 4.4. Let-7a Regulates AKT1 T308 Phosphorylation via PDK1 and PIK3C2A

T308 is located centrally to the AKT1 peptide binding and active sites. Phosphorylation at T308 is necessary and sufficient to activate AKT1 and provide maximal AKT signaling in COS-7 cells [6] and chicken fibroblasts [30]. Indeed, phosphorylation T308 alone is also sufficient for oncogenic transformation [30]. While we found that let-7a transfection significantly depressed T308 phosphorylation in unstimulated cells, EGF stimulation in let-7a supplemented cells leads to a significant increase in AKT1 phosphorylation at T308. Thus, cells with high let-7a levels are more responsive to EGF stimulation at T308 than at S473 relative to control cells. 

We showed that the increased phosphorylation of T308 in stimulated cells supplemented with let-7a results from an increased level of the kinases PDK1 and PIK3C2A that are upstream of AKT1. PIK3C2A is a class II PI3 kinase that is activated in response to EGF and insulin stimulation [52], transducing the signal by converting PIP2 to PIP3. PIP3 is required for binding of PDK1 and AKT1 to the membrane [30] and subsequent activation of AKT1 by phosphorylation at T308 [6] (Figure 1). While PIK3C2A function is not as well understood as those of the class I PI3 kinases, it generally functions in vesicular trafficking and intracellular trafficking [52], with several studies indicating a role in cancer. PIK3C2A mRNA levels are upregulated in activated blastocysts compared to dormant blastocysts [53], indicating a function in actively dividing cells. On the other hand, PIK3C2A expression is inhibited by miR-31 [54] and miR-518A [55], leading to reduced cell proliferation and migration in osteosarcoma cells and gastrointestinal stromal tumors, respectively. We found that PIK3C2A protein levels, but not RNA levels, are significantly increased in stimulated HEK 293T cells transfected with let-7a. Although it is unclear how let-7a positively regulates PIK3C2A abundance, one of the almost 1000 predicted and confirmed let-7a targets [26,27] must control PIK3C2A expression in a yet unrecognized manner. Since PIK3C2A regulation by let-7a remains elusive, identifying the direct let-7a target that in turn regulates PIK3C2A protein levels will form the basis of future work.

In addition to increased PIK3C2A levels, we also observed an increase in the protein level of the AKT1 T308 kinase, PDK1. PDK1 can be positively or negatively regulated by its interaction with several proteins, such as collagen type XI aplha1 (COL11A1), short-form Ron (sfRON), Metadherin (MTDH), tongue cancer resistance-related protein-1 (TCRP1), and the tumor suppressor candidate 4 (TUSC4) [56], yet few data are available regarding the regulation of PDK1 protein abundance. PDK1 is enriched in myelomonocytic acute leukemia patients [57] and regulates cell proliferation in hemangiomas [58]. Interestingly, PDK1 abundance is controlled by the ubiquitin-fold modifier 1 (UFM1), a member of the ubiquitin-like protein (UBL) family, in gastric cancer cells, leading to PDK1 degradation [59]. The UFM1 mRNA does not encode specific let-7a binding sites, but the expression of several other protein degradation pathway components is controlled by let-7a, such as the E3 ubiquitin-protein ligase tripartite motif-containing 71 (TRIM71) [45]. While the mechanism of let-7a control of PDK1 abundance requires further investigation, it is plausible that disruption of PDK1 degradation may lead to increase PDK1 abundance. 

In summary, we observed that high let-7a levels result in an increased abundance of PIK3C2A and PDK1, which magnifies the ability of cells to respond to EGF stimulation, leading to hyperphosphorylated and hyperactive AKT1. 

### 4.5. Expanding Connections between AKT1 and the Let-7 Family

We presented the first evidence of a pathway linking miRNA let-7a levels with AKT1 phosphorylation status, where let-7a also controls the PI3K/AKT cell survival pathway. Several miRNAs are well known to regulate AKT1 abundance, and AKT1, in turn, regulates miRNA abundance via the terminal nucleotidyltransferase TENT2 (Gld2, PAPD4, TUT2). We previously showed that AKT1 negatively regulates TENT2, which in turn regulates let-7a stability [60,61]. The TENT protein family includes members with different RNA-targeting activities and substrate selectivity. TENTs are responsible for 3′-terminal modification of many different miRNA targets [62] with TENT2 stabilizing miRNAs by 3′-terminal mono-adenylation [63]. AKT1 phosphorylates TENT2 at S116, effectively abolishing TENT2 activity and destabilizing miRNAs miR-122 and let-7a [60,61]. 

Interestingly, different let-7 family members fulfill different functions in regulating AKT activity. A previous study showed that insulin-PI3K-mTOR signaling is suppressed by let-7f, decreasing AKT phosphorylation at S473 and T308 when cells were stimulated with insulin [64]. Let-7f transfection directly regulated members of the PI3K-mTOR pathway, including Insulin receptor (INSR), insulin-like growth factor 1 receptor (IGF1R), insulin receptor substrate 2 (IRS2), phosphoinositide-3-kinase interacting protein 1 (PIK3IP1), AKT2, TSC complex subunit 1 (TSC1), and RICTOR [64]. In contrast, our data show that let-7a transfection increases AKT1 phosphorylation at T308 in growth factor-stimulated cells. While the let-7 miRNA family members are highly similar in sequence [65], small changes in the sequence alter target specificity, and let-7a and let-7f were previously shown to assume different biological roles. In immune cells, for example, let-7a is pro-inflammatory by targeting IL-13 [66]. On the other hand, let-7f negatively controls macrophage immune response to *Mycobacterium tuberculosis* infection by targeting A20, a feedback inhibitor of the NF-κB pathway [67]. These different functions of let-7a and let-7f further demonstrate the complexity of the let-7-AKT1 regulatory pathway. Our data suggest that EOC cells require low let-7a levels in adherent cells with hyperphosphorylated AKT, as high let-7a would deregulate AKT1 signaling by altering phospho-status at both sites. Targeting let-7 in EOC cells may thus be exploited to specifically target AKT1-driven cancers and reduce risks of metastasis. 

## 5. Conclusions

In summary, we described the first evidence of regulation of AKT1 at both the protein and phosphoprotein levels by let-7 family mRNAs. In epithelial ovarian cancer cells, increased let-7 miRNA abundance was identified in spheroid cells, implicating a new role for the let-7 family in AKT1-dependent metastasis. We found that the miRNA let-7a controls AKT1 phosphorylation by deregulating PI3K pathway components, including PI3KC2A, PDK1, and RICTOR. Indeed, in cells treated with different let-7 miRNAs, we found AKT1 S473 phosphorylation was insensitive to growth factor stimulation. Let-7a uniquely deregulated AKT1 signaling by enabling hyper-phosphorylation of T308 and hyper-activity in AKT signaling. Together, the data reveal the ability of the let-7 family of miRNAs to regulate the PI3K/AKT pathway in human cells.

## Data Availability

The data presented in this study are available in the main article or Supplementary Material Files.

## Supplementary Materials

The following are available online at https://www.mdpi.com/article/10.3390/cells11050821/s1, Table S1: Oligonucleotides used in this study, Table S2: Antibodies used in this study. Figure S1: AKT1 phosphorylation is not changed in unstimulated cells [26, 27, 68, 69, 70].
