# Supplementary material for: miRNA-Dependent Regulation of AKT1 Phosphorylation
Source: Cells. 2022 Feb 26;11(5):821. doi: 10.3390/cells11050821 (PMC8909289; doi:10.3390/cells11050821)
Supplement: Supplementary file 1 [file cells-11-00821-s001.zip › cells-1595063-supplementary.pdf]

cells MDPI

miRNA-Dependent Regulation of AKT1 Phosphorylation

Mallory I. Frederick [1], Tarana Siddika [1], Pengcheng Zhang [1], Nileeka Balasuriya [1], Matthew A. Turk [1], Patrick O'Donoghue [1,2] and Ilka U. Heinemann [1,*]

[1] Department of Biochemistry, The University of Western Ontario, 1151 Richmond Street, London, ON N6A 5C1, Canada
[2] Department of Chemistry, The University of Western Ontario, 1151 Richmond Street, London, ON N6A 5C1, Canada
* Correspondence: ilka.heinemann@uwo.ca

Supplementary Materials:

Table S1. Oligonucleotides used in this study.

Oligonucleotide (5′-3′)	SOURCE
Synthetic miRNA: let-7a<br>pUGAGGUAGUAGGUUGUAUAGUU	[1,2]
Synthetic miRNA: let-7b<br>pUGAGGUAGUAGGUUGUGUGGUU	[1,2]
Synthetic miRNA: let-7g<br>pUGAGGUAGUAGUUUGUACAGUU	[1,2]
Primer: RT-PCR, let-7a/d/e/f:<br>GTCGTATGCAGAGCAGGGTCCGAGGTATTCGCACTGCATACGACAACTAT	This paper
Primer: RT-PCR, let-7b:<br>GTCGTATGCAGAGCAGGGTCCGAGGTATTCGCACTGCATACGACAACCAC	This paper
Primer: RT-PCR, let-7c:<br>GTCGTATGCAGAGCAGGGTCCGAGGTATTCGCACTGCATACGACAACCAT	This paper
Primer: RT-PCR, let-7g:<br>GTCGTATGCAGAGCAGGGTCCGAGGTATTCGCACTGCATACGACAACTGT	This paper
Primer: RT-PCR, let-7i:<br>GTCGTATGCAGAGCAGGGTCCGAGGTATTCGCACTGCATACGACAACAGC	This paper
Primer: RT-PCR, SNORD47<br>GTCGTATGCAGAGCAGGGTCCGAGGTATTCGCACTGCATACGACAACCTC	[3]
Primer: qPCR, let-7a/b/c, Forward:<br>AGGCTGAGGTAGTAGGTTG	This paper
Primer: qPCR, let-7d, Forward:<br>AGGCAGAGGTAGTAGGTTG	This paper
Primer: qPCR, let-7e, Forward:<br>AGGCTGAGGTAGGAGGTTG	This paper
Primer: qPCR, let-7f, Forward:<br>AGGCTGAGGTAGTAGATTG	This paper
Primer: qPCR, let-7g/i, Forward:<br>AGGCTGAGGTAGTAGTTTG	This paper
Primer: qPCR, SNORD47, Forward:<br>ATCACTGTAAAACCGTTCCA	[3]
Primer: qPCR, let-7 & SNORD47, Reverse:<br>GAGCAGGGTCCGAGGT	[3]
Primer: qPCR, PIK3C2A, Forward:<br>TGAATAGTTCATTAGTGCAATTCCTT	[4]
Primer: qPCR, PIK3C2A, Reverse:	[4]

	GGCATCTTTGAGAAGCCAAT	
	Primer: qPCR, GAPDH, Forward: GAAGGTGAAGGTCGGAGTCAAC	[5]
	Primer: qPCR, GAPDH, Reverse: CAGAGTTAAAAGCAGCCCTGGT	[5]

Table S2. Antibodies used in this study.

ANTIBODY	SOURCE	IDENTIFIER
anti-pan-AKT	Cell Signaling Technology	Cat#2920; RRID:AB_1147620
anti-AKT1	Cell Signaling Technology	Cat#2938; RRID:AB_915788
anti-AKT2	Cell Signaling Technology	Cat#3063; RRID:AB_2225186
anti-AKT3	Cell Signaling Technology	Cat#4059; RRID:AB_2225351
anti-pAKT^{T308}	Cell Signaling Technology	Cat#9275; RRID:AB_329828
anti-pAKT^{S473}	Cell Signaling Technology	Cat#9271; RRID:AB_329825
anti-GAPDH	Millipore	Cat#MAB374; RRID:AB_2107445
anti-GSK-3α/b	Santa Cruz Biotechnology	Cat#sc-7291; RRID:AB_2279451
anti-pGSK-3α/b	Cell Signaling Technology	Cat#9331; RRID:AB_329830
anti-PDK1	Cell Signaling Technology	Cat#3062; RRID:AB_2236832
anti-pPDK1	Cell Signaling Technology	Cat#3438; RRID:AB_2161134
anti-PP2A	BD Biosciences	Cat#610555; RRID:AB_397909
anti-PIK3C2A	Cell Signaling Technology	Cat#12402; RRID:AB_2797900
anti-RICTOR	Bethyl Laboratories	Cat#A300-459A; RRID:AB_2179967
anti-mTOR	Cell Signaling Technology	Cat#2972; RRID:AB_330978
anti-vinculin	Cell Signaling Technology	Cat#13901; RRID:AB_2728768

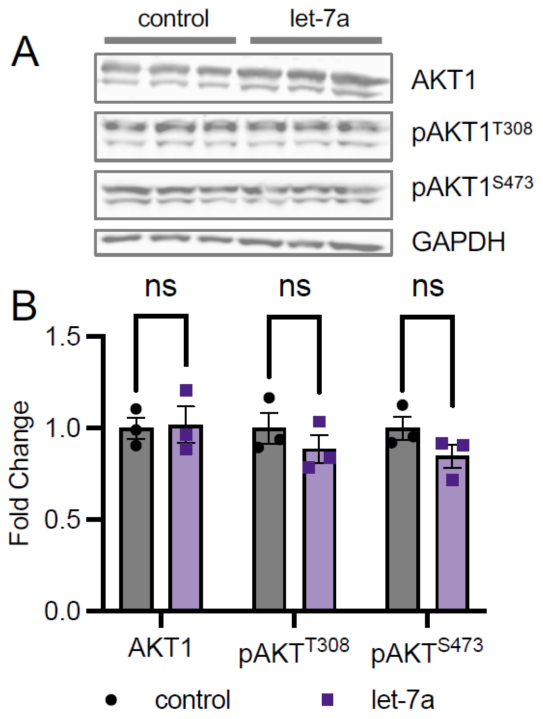

Figure S1. AKT1 phosphorylation is not changed in unstimulated cells. (A) Western blots and (B) quantification of mCherry-AKT1 and mCherry-pAKT1 levels in HEK 293T cells co-transfected with 5 μg mCherry-mAKT1 plasmid and 60 fmol let-7a (purple) or a control lacking RNA (black) for 24 hours. The data include N = 3 biological replicates. Error bars show ± 1 SEM. ns = not significant.

References

1. Chen, Y.; Wang, X. miRDB: An online database for prediction of functional microRNA targets. *Nucleic Acids Res.* **2020**, *48*, D127–D131. https://doi.org/10.1093/nar/gkz757.
2. Liu, W.; Wang, X. Prediction of functional microRNA targets by integrative modeling of microRNA binding and target expression data. *Genome Biol.* **2019**, *20*, 18. https://doi.org/10.1186/s13059-019-1629-z.
3. Naderi, M.; Abdul Tehrani, H.; Soleimani, M.; Shabani, I.; Hashemi, S.M. A home-brew real-time PCR assay for reliable detection and quantification of mature miR-122. *Appl. Immunohistochem. Mol. Morphol.* **2015**, *23*, 601–606. https://doi.org/10.1097/PAI.0000000000000125.
4. Boukhalfa, A.; Nascimbeni, A.C.; Ramel, D.; Dupont, N.; Hirsch, E.; Gayral, S.; Laffargue, M.; Codogno, P.; Morel, E. PI3KC2alpha-dependent and VPS34-independent generation of PI3P controls primary cilium-mediated autophagy in response to shear stress. *Nat. Commun.* **2020**, *11*, 1–9. https://doi.org/10.1038/s41467-019-14086-1.
5. Barber, R.D.; Harmer, D.W.; Coleman, R.A.; Clark, B.J. GAPDH as a housekeeping gene: analysis of GAPDH mRNA expression in a panel of 72 human tissues. Physiol. Genom. 2005, 21, 389–395, doi:10.1152/physiolgenomics.00025.2005.
